# A renewed perspective on TCA intermediate cis-aconitate: mechanisms and therapeutic options against influenza virus

**DOI:** 10.1038/s44321-026-00380-1

**Published:** 2026-02-16

**Authors:** Claudia Claus, Igor Kovacevic

**Affiliations:** 1 Institute of Medical Microbiology and Virology, Medical Faculty, Leipzig University, Leipzig, Germany; 2 Institute of Physiological Chemistry, Medical Faculty, Martin Luther University Halle-Wittenberg, Halle (Saale), Germany

**Keywords:** Metabolism, Microbiology, Virology & Host Pathogen Interaction

## Abstract

C. Claus and I. Kovacevic discuss the identification of cis-aconitate, a mitochondria-derived metabolite, with dual antiviral and anti-inflammatory activity against influenza, as reported by M. Si-Tahar and colleagues, in this issue of *EMBO Mol Med*.

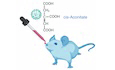

## Teaser

Effective therapeutic strategies for influenza, ranging from seasonal flu outbreaks to sporadic pandemics, should ideally combine antiviral efficacy to accelerate viral clearance with targeted immunomodulation to mitigate excessive inflammation. In this issue of *EMBO Molecular Medicine*, Cezard et al introduce the inhibitory potential of the tricarboxylic acid (TCA) cycle intermediate cis-aconitate against influenza virus. Cis-aconitate is promising for further translational evaluation, as it meets the dual targeting criteria of antiviral and anti-inflammatory activity at both early as well as late stages of infection. Remarkably, these effects occur independently of its conversion into itaconate, a well-characterized immunomodulatory TCA derivative. This novel aspect of cis-aconitate further supports continued consideration of TCA cycle intermediates beyond their canonical metabolic roles.

**Figure 1:**
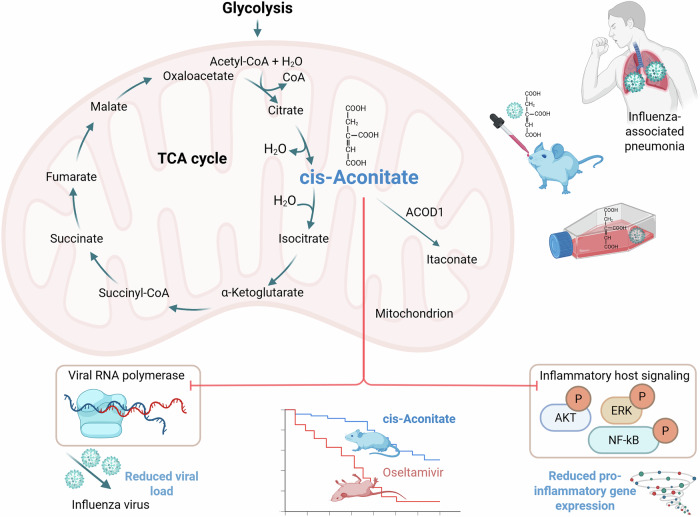
The dual antiviral and anti-inflammatory properties of cis-aconitate emphasize its therapeutic potential. In an influenza virus-infected mouse model, Cezard et al show that cis-aconitate administration reduced viral load in the lungs, attenuated inflammatory cytokine production and immune cell infiltration, and significantly improved survival from pneumonia-induced death. Notably, cis-aconitate remained effective even at later infection stages: 50% of mice infected with a lethal dose of influenza virus and treated at day 4 post-infection survived, compared with 0% survival in those receiving the approved antiviral oseltamivir. The figure was created using BioRender.com

Viruses, and particularly RNA viruses, are characterized by high mutation rates that could lead to adaptation to new hosts during spillover events, and to the emergence of beneficial mutations that support, for example, evasion from pre-existing immunity against earlier circulating viruses. This genetic plasticity also accelerates the development of resistance to antiviral therapeutics. The influenza virus is a well-known and highly relevant example of these adaptive processes. Continuous mutations within its genome and the resulting evasion of host immune responses drive annual seasonal epidemics and facilitate the emergence of occasional pandemics. Beyond the substantial disease burden of seasonal influenza, there is a persistent threat posed by the potential adaptation of highly pathogenic avian influenza A viruses to human hosts. Furthermore, the occurrence of mutations can also reduce the effectiveness of antiviral drugs used against influenza viruses (Bonomini et al, 2025). In addition to the risk of selecting resistance-associated mutations, the timing of anti-influenza treatment with approved direct-acting antivirals (DAAs) that target viral components is crucial and should be initiated within 48 h after the onset of symptoms. A widely used and standard of care for both influenza virus prophylaxis and treatment is oseltamivir (trade name Tamiflu®) (Bonom**i**ni et al, 2025). As a sialic acid analog, oseltamivir inhibits the influenza neuraminidase, which is a surface glycoprotein and an exosialidase essential for the efficient release of new virions from infected cells. Besides DAAs, host-targeted antivirals (HTAs) represent a growing area of research aimed at providing robust and broad-spectrum antiviral therapeutic options. These drugs target host cell factors required for influenza replication or contributing to pathogenesis. Currently, there is a growing interest in the potential of HTAs as a complementary approach to DAAs. Several HTA candidates have progressed to clinical trials, and novel agents continue to be identified and evaluated; for instance, in vitro data demonstrated the potent antiviral activity of the MEK1/2 (mitogen-activated protein kinase 1 and 2) inhibitor zapnometinib against highly pathogenic avian influenza A viruses (Bonom**i**ni et al, 2025; Schreiber et al, 2025). Generally, combining DAAs with HTAs yields superior clinical outcomes compared to monotherapy (Bonom**i**ni et al, 2025). Consistent with this, the co-administration of zapnometinib with two distinct DAAs demonstrated synergistic antiviral effects (Schre**i**ber et al, 2025). In their current study, Cezar et al provide compelling evidence for cis-aconitate as a novel compound against influenza viruses. Their findings suggest that cis-aconitate is a promising candidate for further therapeutic development due to its dual-action profile: it exerts both potent antiviral activity by inhibiting viral replication and anti-inflammatory effects by modulating the host immune response (Fig. 1).

The role of cis-aconitate in inflammatory conditions, including viral infections, and its immunomodulatory properties, have been so far mainly evaluated based on its conversion to itaconate by the enzyme ACOD1 (cis-aconitate decarboxylase 1). Itaconate itself is an important immunoregulatory intermediate with potent anti-inflammatory activity and thus offers treatment options for various medical conditions with inflammatory involvement, including virus infections such as influenza (Gao et al, 2025). Activation of immune cells through different stimuli, including virus infections, leads to enhanced expression of ACOD1, and as such, to diversion of cis-aconitate to itaconate (Wu et al, 2020). An upregulation of ACOD1 was also present in all experimental models used by Cezard et al for influenza virus infection. While the immunoregulatory actions of itaconate have been extensively characterized, the role of cis-aconitate was usually confined to being a precursor of itaconate. In contrast, the effects of cis-aconitate during influenza virus infection were independent of its conversion to itaconate, as they persisted in ACOD1-deficient mice and cell lines infected with influenza virus. Moreover, while some properties are shared between these two compounds, the underlying mechanisms activated by cis-aconitate appear to be separate from and in parts even opposite to those associated with itaconate. By demonstrating its independence from itaconate, Cezard et al highlight that even well-known metabolites can be promising novel therapeutic candidates with a so far hidden potential.

The protective effects of cis-aconitate involved direct inhibition of viral RNA polymerase and attenuation of host inflammatory signaling pathways, including ERK (extracellular signal-regulated kinases), AKT (protein kinase B), and NF-κB (nuclear factor kappa-light-chain-enhancer of activated B cells) activation. Cezard et al validated their findings across multiple models with relevance to influenza pathogenicity in humans: in vitro in bronchial epithelial cell lines, ex vivo in primary lung epithelial cells and human organotypic lung cultures, and in vivo in a mouse model of influenza virus infection. Importantly, the anti-influenza activity of cis-aconitate was sustained throughout both early and late stages of infection (Fig. 1). This indicates a prolonged therapeutic time window. Such an extension is highly clinically relevant, reflecting the typical time lag in human patients between initial symptom onset and progression to severe symptoms requiring hospitalization.

Both itaconate and cis-aconitate are linked to the tricarboxylic acid (TCA) cycle, also known as the Krebs or citric acid cycle. This further emphasizes the potential of TCA cycle intermediates as antiviral treatment options. The TCA cycle is a central metabolic pathway located within the mitochondrial matrix. Its primary function is the complete oxidation of acetyl-CoA, derived from the catabolic breakdown of glucose and fatty acids, to produce reducing equivalents for ATP generation via oxidative phosphorylation. The TCA cycle is interconnected with carbohydrate, lipid, and amino acid pathways, and as such, has a central role in cellular metabolism. This renders the TCA cycle and its intermediates key targets for viral manipulation, whereby virus-induced metabolic reprogramming creates an environment favorable for efficient viral replication (Sanchez-Garcia et al, 2021). Some TCA intermediates, such as succinate and fumarate, in addition to itaconate, as a TCA derivative, have emerged as key immunomodulatory molecules collectively termed immunometabolites (Tada et al, 2025). In a preceding study, Si-Tahar's group together with colleagues started their investigation into the role of TCA intermediates, as antivirals against influenza viruses, with succinate (Guillon et al, 2022). Their findings showed that intranasal application of succinate reduced the viral load in influenza virus-infected mice and protected the animals against virus-induced pneumonia. Mechanistically, lysine succinylation, as a succinate-directed posttranslational modification of the viral nucleoprotein, interfered with the virus replication cycle through an impaired assembly and trafficking of the viral ribonucleoprotein (vRNP) complexes. These are formed through the association of the viral RNAs with nucleoprotein molecules. Cezard et al (2026) systematically extended this past work in their current study, evaluating the effects of eight TCA cycle metabolites on influenza virus infection in human bronchial epithelial cells. Notably, the authors observed that, unlike succinate, cis-aconitate exhibited efficacy against both influenza A and B viruses, the two types of influenza viruses that cause seasonal epidemics in humans. Furthermore, its mechanism was not restricted to antiviral activity; it also significantly attenuated the host pro-inflammatory response. In addition, and in contrast to succinate, cis-aconitate reduced the pro-inflammatory response after stimulation with polyinosinic:polycytidylic acid (poly (I:C)), as a synthetic toll-like receptor-3 (TLR3) agonist and a mimetic of the RNA virus replication intermediate dsRNA.

The observations by Cezard et al highlight cis-aconitate’s dual efficacy against influenza viruses, even in an advanced infection phase, where current therapies, including oseltamivir, are often ineffective (Fig. 1). With their comprehensive study design involving advanced human lung organotypic models and murine models of influenza infection, Cezard et al established the foundation for further translational evaluation of cis-aconitate. As the authors emphasize, a follow-up investigation is required to fully elucidate the mechanism of action and characterize the pharmacokinetics of cis-aconitate. In addition, the assessment of the therapeutic efficacy against a broader range of influenza viruses, including avian influenza A viruses, would further support our preparedness for future influenza outbreaks and pandemics. Moreover, exploring combination therapies with DAAs or HTAs is essential to identify potential drug-drug interactions and to determine additive or synergistic effects. Such comprehensive characterization will be instrumental in establishing cis-aconitate as a robust addition to the HTA repertoire for influenza management.
